# Association study in the 5q31-32 linkage region for schizophrenia using pooled DNA genotyping

**DOI:** 10.1186/1471-244X-8-11

**Published:** 2008-02-25

**Authors:** Irina Zaharieva, Lyudmila Georgieva, Ivan Nikolov, George Kirov, Michael J Owen, Michael C O'Donovan, Draga Toncheva

**Affiliations:** 1 Department of Medical Genetics, Medical University Sofia, 2 Zdrave St, 1431 Sofia, Bulgaria; 2 Department of Psychological Medicine, School of Medicine, Cardiff University, Heath Park, Cardiff CF14 4XN, UK

## Abstract

**Background:**

Several linkage studies suggest that chromosome 5q31-32 might contain risk loci for schizophrenia (SZ). We wanted to identify susceptibility genes for schizophrenia within this region.

**Methods:**

We saturated the interval between markers D5S666 and D5S436 with 90 polymorphic microsatellite markers and genotyped two sets of DNA pools consisting of 300 SZ patients of Bulgarian origin and their 600 parents. Positive associations were followed-up with SNP genotyping.

**Results:**

Nominally significant evidence for association (p < 0.05) was found for seven markers (D5S0023i, IL9, RH60252, 5Q3133_33, D5S2017, D5S1481, D5S0711i) which were then individually genotyped in the trios. The predicted associations were confirmed for two of the markers: D5S2017, localised in the *SPRY4-FGF1 *locus (p = 0.004) and IL9, localized within the IL9 gene (p = 0.014). Fine mapping was performed using single nucleotide polymorphisms (SNPs) around D5S2017 and IL9. In each region four SNPs were chosen and individually genotyped in our full sample of 615 SZ trios. Two SNPs showed significant evidence for association: rs7715300 (p = 0.001) and rs6897690 (p = 0.032). Rs7715300 is localised between the *TGFBI *and *SMAD5 *genes and rs6897690 is within the *SPRY4 *gene.

**Conclusion:**

Our screening of 5q31-32 implicates three potential candidate genes for SZ: *SMAD5*, *TGFBI *and *SPRY4*.

## Background

Schizophrenia (SZ) is a common, severe and disabling disorder that in most cases requires a long-term medical and social care. The lifetime risk for SZ in the population worldwide is around 1%. Family, adoption and twin studies have shown conclusively that a genetic component plays the most important role in its aetiology [1]. At present, the number of susceptibility loci, the disease risk conferred by each locus and the degree of interaction between them remain unknown [2]. The mode of transmission is complex and non-Mendelian and is probably contributed by a small number of genes of moderate effect, or by many genes of small effect, or a mixture of the two [3].

**Figure 1:**
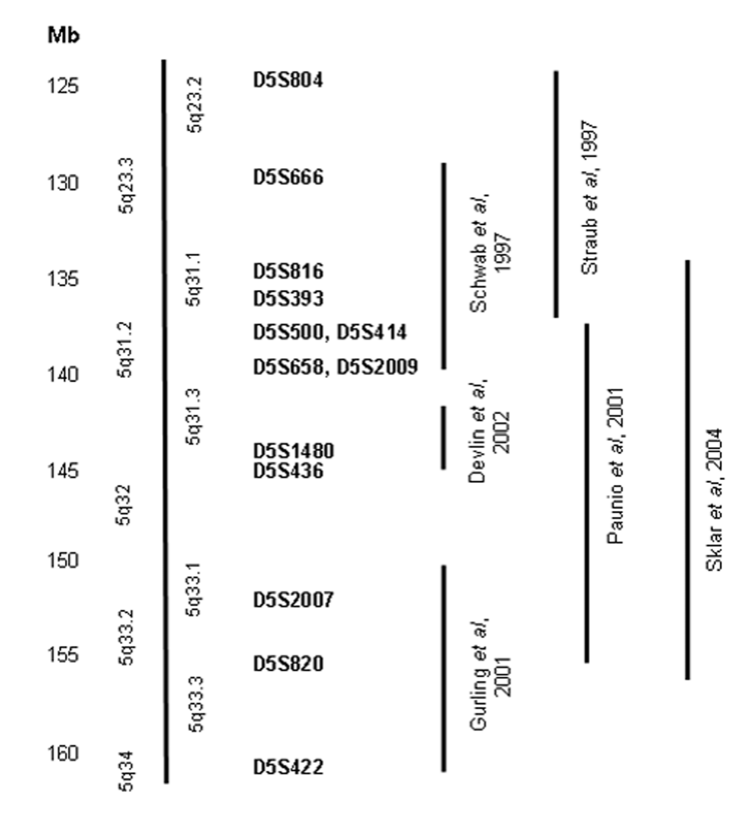
**Linkage findings in schizophrenia in the 5q23-33 region**. Straub *et al *(1997) [5] reported a maximum LOD score of 3.04 for schizophrenia at D5S393. Schwab *et al *(1997) [6] reported maximum LOD score of 1.8 for the marker IL9 by two point lod score analysis. Paunio *et al *(2001) [7] obtained a maximum lod score of 3.56 at D5S414. Gurling *et al *(2001) [50] reported a LOD score 3.6 at D5S422. Devlin *et al *(2002) [8] reported a LOD score of 3.4 at D5S1480 and Sklar *et al *(2004) [9] obtained a NPL score of 3.09 (p = 0.0012) at D5S820.

In the present study we investigated one strong region of linkage to schizophrenia: 5q31-32. This region emerged as one of the five most consistent regions in a meta-analysis of the genome-wide linkage studies [4]. The main linkage findings for 5q23-33 are summarised in Figure 1.

We concentrated upon a minimal region of interest between markers D5S666 and D5S436 as it includes five of the regions showing linkage to schizophrenia [5-9]. We decided to concentrate on this region, rather than on the full region, as it is the most likely region to contain susceptibility genes, due to the concentration of five linkage findings. We would have been unable to provide similar dense coverage of the whole interval with the funding we received for this project. This interval is ~14 Mb long and contains ~330 genes (UCSC built 35, May 2004), of which 52 constitute the protocadherin α, β, γ clusters. Protocadherins are expressed throughout the nervous system and are involved in synapse formation, specification and maintaining, which make them potential candidate genes for schizophrenia. This group of genes and their relation to schizophrenia has being investigated by several research groups [10-13]. Other promising candidate genes in this region are *NRG2 *(Neuregulin 2) and *IL9 *(P40 cytokine). The neuregulins are a family of growth and differentiation factors with a wide range of functions in the nervous system [14]. Neuregulin signalling plays an important role in many neurological disorders including multiple sclerosis, traumatic brain and spinal cord injury, peripheral neuropathy, and possibly schizophrenia [14-16]. According to the glial growth factors deficiency and synaptic destabilization hypothesis of SZ, functional deficiency of glial growth factors and of growth factors such as neuregulin, insulin-like growth factor I, insulin, epidermal growth factor, neurotrophic growth factors, erbB receptors and others, are among the distal causes in the genotype-to-phenotype chain leading to the development of SZ [17]. Cytokines are key molecules regulating immune/inflammatory reactions. They are involved in brain development, regulation of dopaminergic and GABAergic differentiation, and synaptic maturation. Certain cytokines are postulated to have a central role in the neurodevelopmental defects in SZ [18,19].

The systematic association analysis of complex disorders requires genotyping of numerous genetic markers over particular genomic regions, or more recently the entire genome, in large samples. The cost of such studies is prohibitive for most laboratories. DNA pooling is a way to decrease the cost, time and labour that are involved in a large-scale genotyping [20]. Briefly, in DNA pooling equimolar amounts of DNA are taken from each individual mixed to form two sets of pools, cases and controls. Predicted allele frequencies are then estimated on the basis of the intensities produced in each pool. DNA pooling is capable of detecting loci with small effect sizes and decreases the cost of the analysis by orders of magnitude. The power of pooling studies is approximately the same as for individual genotyping of affected and non-affected individuals, with a mean error rate of pooled analysis reported for different pooling techniques in the region of <2% [20-23].

In the present study, the initial DNA pooling and individual genotyping was performed using microsatellite markers, and fine mapping was performed with single nucleotide polymorphisms (SNPs). We reasoned that microsatellite markers have several advantages over SNPs for initial screening: they are highly polymorphic with corresponding high degree of heretozygosity (on average ~70%) and they can detect linkage disequilibrium (LD) over larger distances than SNPs (~100 kb range compared to ~30 kb range for SNPs) [24,25] probably due to the fact that they have a high mutation rate, making it possible for certain of their alleles to capture associations of more recent origin. Therefore, we reasoned that we could cover the 14 Mb interval with fewer microsatellite markers, than with SNPs, thus making it possible to conduct the study within the budget of our research project.

We chose to use an initial screening step of applying pooled analysis to polymorphic microsatellite spanning 5q31-32, and a follow up stage of individual genotyping in the same samples from which the pools were constructed for markers showing nominally significant evidence for association (p < 0.05). SNPs in regions containing a confirmed nominally significant microsatellite marker (p < 0.05) were then examined further for association with SNPs in order to refine the position of the association signal.

## Methods

### Clinical sample

For the initial screen, we used two parent-proband DNA pools: one from 300 SZ patients and the second one from their 600 parents. All trios are of Bulgarian origin. They were either inpatients from five different psychiatric hospitals, or outpatients from four of the largest psychiatric dispensaries in Bulgaria. All had a history of hospitalization for a schizophrenic episode. Each proband was interviewed with an abbreviated version of the SCAN instrument (Schedules for Clinical Assessment in Neuropsychiatry) [26]. Consensus best-estimate diagnoses were made according to DSM-IV criteria (*Diagnostic and Statistical Manual of Mental Disorders*, 4th edn.1994) [27] by two raters using information from the interview and hospital notes. All patients included in the study met DSM-IV criteria for schizophrenia. Local ethics committee approval was obtained from all the regions where patients were recruited. All patients and their parents were given information sheets and provided written informed consent for participation in genetic association studies. DNA was extracted by standard phenol-chloroform method from peripheral blood. The selected microsatellite markers for individual genotyping were genotyped in the same sample of 300 trios families that were included in the pools, while the SNPs were genotyped in the full sample of 615 SZ trios that have been recruited by the team in Bulgaria. To inform our choice of SNPs for fine-mapping, we used data from another pooling study on 574 SZ trios (explained in more detail below). This larger pool includes the original pool of 300 trios used in the main microsatellite study, and a second pool of 274 trios from our full sample of 615 trios.

### Choice of markers

#### Microsatellites

Microsatellite markers were selected from JBIRC database [28], developed on the basis of analysis of microsatellite markers covering the whole human genome [25], and the UCSC Genome Browser (built 35, May 2004) [29]. We aimed to place markers at an average density of one every ~100 kb. We first selected validated polymorphic markers within our region of interest using all available information from JBIRC. In cases where the distance between validated markers was greater than 100 kb, we selected non-validated di-, tri- tetra- and penta-nucleotide repeats from UCSC and validated them ourselves by examining whether they were polymorphic. We selected a total of 140 microsatellite markers from the two databases. 50 markers were not included in the analysis because they produced heavy stutter bands or were not polymorphic. We successfully analysed 90 markers, of which 24 were chosen from JBIRC and 66 from UCSC database (39 are novel markers that have not been validated before). The average distance between the analysed 90 polymorphic markers was ~150 kb and 72% of the microsatellite markers were di-nucleotide repeats, the remaining were tri-, tetra- or penta-nucleotide repeats.

#### SNPs

In order to improve our chances of identifying the most significant SNPs around significant microsatellites for the fine mapping, we selected SNPs from the pooling data of a genome-wide association (GWA) study of schizophrenia in the same Bulgarian trios, conducted by the Department of Psychological Medicine, Cardiff University towards the end of the microsatellite project (manuscript in press) [30]. The results of the study on Illumina arrays became available just at the time when we started selecting SNPs for fine mapping. We wanted to saturate an extended area of ~400 kb around positive microsatellites, in order to identify the peak of the maximum significance in each region. The latest versions of the HapMap database contain hundreds of SNPs in these regions, making it impossible for us to provide dense coverage with the funding available. This is why we decided to make use of our Illumina data. We reasoned that SNPs which are not significantly associated in that study of overlapping samples, were most likely to remain negative after individual genotyping, therefore we could only target those SNPs that produced significant results (at a predicted p-value of ≤ 0.05). The GWA pooling study was carried out with Illumina HumanHap550 Genotyping BeadChip technology using pooled DNA from 574 SZ patients and a pool from all the parents of the cases [30]. The pool of 574 trios includes the pool of 300 trios prepared for the microsatellite association study, plus an additional pool of 274 SZ trios that was prepared from the available SZ trios recruited by our team in Bulgaria. Although the complete number of trios available for individual genotyping was 615, only 574 trios are included in the pools, for the following reasons. 1) A small number of samples had lower DNA concentration, making them unsuitable for pooling. 2) A number of families are multiply affected, providing more than one trio for the analysis of individual genotyping. However, only one trio per family was included in pools.

### Genotyping

For microsatellites we designed flanking primers labelled at their 5' end with a fluorescent dye. PCR primers were designed with the Primer 3 program [31]. All primers were checked with BLAT in order to make sure their sequences were unique. Fragments were amplified using a standard PCR touch-down protocol on thermal cycler DNA Engine Tetrad machines (MJ Research, USA). Fragments were separated on an ABI3100 capillary sequencer (Applied Biosystems, USA). Microsatellite markers for individual genotyping were amplified in a multiplex PCR reaction using the same protocol as above.

SNPs were genotyped using the Sequenom MassARRAY™ iPlex™ chemistry (Sequenom, San Diego, California, USA) [32] or Amplifluor™ SNP Genotyping Systems (Serologicals Corporation, USA) [33,34] according to the recommendations of the manufacturers.

### Pooled DNA genotyping

The principles of pooled DNA genotyping have been described before [21,22]. Briefly, an equimolar amount of DNA was taken from every individual from one sample set and put into a single tube (one pool). The DNA from every sample was quantified using the PicoGreen ds DNA Quantification Reagent (Molecular Probes, Eugene, Oregon, USA). Each pooled DNA was amplified in triplicate and the PCR products were separated on an ABI3100 capillary sequencer. If any of the replicates gave more than 3% difference in any one allele, the experiment was repeated, or this replicate excluded. The peak heights of the signal representing each of the alleles were measured using GENOTYPER 2.5 software. The peak heights were used to estimate the relative allele frequencies in the pools, assuming that peak height is directly proportional to the concentration of that allele in the pool. Thus, the allele frequencies were estimated from the peak height for each allele divided by the sum of the peak heights for all alleles. We didn't apply correction for stutter bands and differential amplification, as described before [21].

### Statistical analysis

For analysis of pooling results we used the CLUMP program [35] to compare the predicted allele frequencies of probands with the non-transmitted parental alleles (our pseudo-controls). For each marker we ran 1000 simulations and estimated the nominal p-value. Individual genotyping results were analyzed with the Extended Transmission Disequilibrium Test (ETDT) for multiallelic markers and the Transmission Disequilibrium Test for SNPs [36-38]. Analyses of linkage disequilibrium (LD) between SNPs (*r*^2 ^and *D*') were performed using Haploview [39,40].

## Results

### Microsatellites

We designed and genotyped 140 microsatellite markers covering a region of ~14 Mb (between markers D5S666 and D5S436, Figure 1) in parents and probands pools. After excluding non-polymorphic markers and those producing heavy stutter bands, we successfully analysed 90 polymorphic microsatellite markers. Seven markers showed suggestive evidence for association with SZ (p < 0.05) and were selected for individual genotyping. These were D5S0023i (p = 0.033), IL9 (p = 0.046), RH60252 (p = 0.041), 5Q31-33_33 (p = 0.031), D5S2017 (p = 0.013), D5S1481 (p = 0.030), D5S0711i (p = 0.028). In order to confirm the results from the pooling experiment, we performed individual genotyping of these markers in the sample of 300 parent-proband trios that were included in the pools. Results from individual genotyping are presented in Table 1. Marker D5S0023i could not be analyzed as it produced heavy stutter bands, making allele calling unreliable. Two markers remained nominally significant after the individual genotyping: marker IL9 (p = 0.014), located within the IL9 (interleukin 9 precursor) gene and marker D5S2017 (p = 0.004), located 28.5 kb 3' from the SPRY4 (Sprouty homolog 4) gene. The distance between the two markers is approximately 6.5 Mb.

**Table 1:** Pooling and individual genotyping results for microsatellite markers showing suggestive evidence for association.

**#**	**Marker name**	**Position in bp (UCSC May, 04)**	**Repeat type**	**Number of repeats**	**Pooling p-value**	**Individual genotyping p-value**
1	D5S0023i	chr5:133235407–133235452	AAGG	9	0.033	no data
2	IL9	chr5:135256322–135256367	AC	12	0.046	0.014
3	RH60252	chr5:139324127–139324160	AC	11	0.041	0.51
4	5Q31-33_33	chr5:141503743–141503777	TAA	6	0.031	0.10
5	D5S2017	chr5:141713613–141713660	TG	6	0.013	0.004
6	D5S1481	chr5:144559890–144559927	TAT	10	0.030	0.77
7	D5S0711i	chr5:145722795–145722839	AAACA	9	0.028	0.99

### SNPs

**Figure 2:**
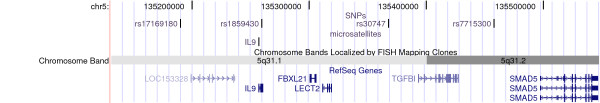
**Markers and genes in the region around IL9 marker**. Included are base pair position according to UCSC built 35, May 2004, chromosome band, gene and the genotyped SNPs and microsatellite markers within the region.

**Figure 3:**
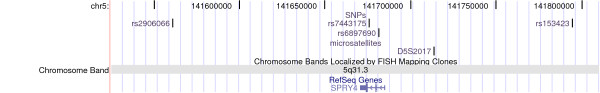
**Markers and genes in the region around D5S2017 marker**. Included are base pair position according to UCSC built 35, May 2004, chromosome band, gene and the genotyped SNPs and microsatellite markers within the region.

In order to further investigate the two candidate regions, we performed fine mapping using SNPs. Eight SNPs were selected from regions of 400 kb surrounding the microsatellites D5S2017 and IL9 from the data of our GWA study in 574 SZ trios carried out in the same population (see Methods). These eight SNPs had shown suggestive significance for association (p = 0.05) in the pooling data. Rs17169180, rs1859430, rs30747 and rs7715300 are around the IL9 area, and rs2961720, rs7443175, rs6897690 and rs153423 are from the D5S2017 area (Figures 2 and 3). We could not produce a robust assay for genotyping rs2961720, so we substituted it with rs2906066 which was in complete LD with rs2961720 (*r*^2 ^= 1) in the CEU trios sample (Utah residents with ancestry from Northern and Western Europe) from HapMap [41]. Results from the individual genotyping are presented in Table 2.

**Table 2:** Summarized individual genotyping results for SNPs and TDT results.

**SNP ID**	**Position**	**Gene name**^**▲**^	Type*	**Pooling p-value**	**Total sample (N trios)**	**Frequency children**	**Frequency parents**	**T/NT**	**χ**^**2**^	**Individual genotyping total sample p-value**	**Individual genotyping 300 trios pool p-value**
rs17169180	135188954	LOC153328	C/A	0.008	613	0.14	0.15	142/176	3.64	0.06	0.20
rs1859430	135258412	IL9	T/C	0.04	599	0.19	0.20	177/203	1.78	0.18	0.14
rs30747	135366739	TGFBI	C/A	0.04	590	0.05	0.05	60/58	0.03	0.85	0.28
rs7715300	135456919	TGFBI	C/A	0.03	612	0.06	0.08	64/108	11.26	0.001	0.18
rs2906066	141560461	-	T/C	0.05	589	0.14	0.13	122/145	1.98	0.16	0.57
rs7443175	141675612	SPRY4	T/C	0.03	612	0.23	0.25	239/200	3.46	0.06	0.36
rs6897690	141681136	SPRY4	A/G	0.008	615	0.22	0.24	255/209	4.56	0.03	0.0006
rs153423	141794129	-	C/T	0.05	611	0.21	0.20	180/202	1.27	0.26	0.46

^▲ ^Gene names are given for the intragenic SNPs and SNPs within 30 kb of a gene (column 3,"Gene name").

* Minor allele is always presented first and its frequencies in children and in parents are given in columns 7 and 8 ("Frequency children", "Frequency parents"). Transmission/non-transmission counts from heterozygous parents for the minor allele in each case are in column 9 ("T/NT"). P-values from Transmission Disequilibrium Test (TDT) for the total sample and the pool sample are given in the column 11 ("Individual genotyping total sample p-value") and column 12 ("Individual genotyping 300 trios pool p-value"). Predicted pooling p-values are presented in column 5, ("Pooling p-value"). Pooling p-value is also provided for rs2906066, although it is not on the array, because it is in complete LD with rs2961720 which produced that p-value. All markers were in Hardy-Weinberg equilibrium.

Individual genotyping in the total sample of 615 parent-proband trios confirmed one SNP in each region as significantly associated with SZ: rs7715300 (p = 0.001) and rs6897690 (p = 0.032). One more SNP in each region approached significance (p = 0.06). Rs7715300 is located between two genes: 30 kb 3'of *TGFBI *(transforming growth factor, beta-induced, 68 kDa) and 39.5 kb 5' of *SMAD5 *(SMAD, mothers against DPP homolog 5). Rs6897690 is located in intron1 of *SPRY4 *(sprouty homolog 4). All markers were in Hardy-Weinberg equilibrium. No significant linkage disequilibrium (LD) between the studied SNPs was observed (*r*^2 ^< 0.1). In order to provide the full information, we also present in the Table the p-values provided by the 300 trios in the original pools used for the microsatellite study, which demonstrate some fluctuation from those in the full sample.

## Discussion

Pooling is a fast and cost-effective approach used for systematic screening of complex disease associations, where many markers need to be genotyped in large samples. The power of pooling is approximately the same as individual genotyping and has proved to be an accurate method for detecting allele differences using microsatellite markers or SNPs, with a mean error of <2% for the pooled analysis [21-23,42,43].

In the present study we wanted to investigate the chromosomal region 5q31.1-q32 between markers D5S666 and D5S436 because it includes five reported linkage regions for SZ [5-9] and a number of good candidate genes. We started with saturating the 14 Mb region with microsatellite markers using DNA pooling, an approach proven successful for other complex diseases [25,44,45]. Based on the knowledge that the average length of LD around microsatellite markers is approximately 100 kb, we covered the region with 140 microsatellite markers of which 90 turned out to be polymorphic and gave reliable traces. We found a suggestive evidence for association with SZ for seven markers. Individual genotyping confirmed two of them to be significantly associated: D5S2017 (p = 0.004) and IL9 (p = 0.014). Marker IL9 is located within the IL9 gene and is the same microsatellite previously reported by Schwab *et al *[6] to produce a max LOD score of 1.8 in 14 SZ pedigrees.

We then performed fine mapping with SNPs within an area of ~400 kb surrounding the IL9 and D5S2017 microsatellites. In order to improve our chances of identifying significant association, we chose to genotype only promising SNPs from another pooling study in SZ on Illumina HumanHap550 arrays [30]. That study used an extended sample of 574 Bulgarian trios and was finished just at the time when we were selecting SNPs to follow-up (all 300 trios used in the microsatellite stage of the study were part of that larger sample). We selected 8 SNPs (4 for each region), that had shown nominal significance (p = 0.05) in pools hybridised on Illumina arrays. This considerably reduced the cost of our project, as the two regions contained nearly 200 SNPs on the Illumina arrays (and even more in the HapMap database). Individual genotyping of the SNPs in the full 615 parent-proband trios confirmed the pooling results for two SNPs: rs7715300 (p = 0.001) and rs6897690 (p = 0.032). SNPs rs17169180 and rs7443175 showed only trends toward association (p = 0.06). To show the validity of the pooling approach, we also report data on the 300 trios in our original pools in the Table 2. However our aim was to identify susceptibility loci for SZ which is clearly best achieved by genotyping as large a sample as possible, therefore the full data on the 615 trios constitutes our primary analysis.

Clearly, the strongest signal identified in our study is at rs7715300, located between two genes: 30 kb 3'of *TGFBI *and 39.5 kb 5' of *SMAD5 *(Figure 2). We cannot speculate whether rs7715300 is a causal variant itself, or it is in LD with a marker within any of the two genes (however no SNP within the two genes is in high *r*^2 ^with rs7715300). A Bonferroni correction for 90 microsatellite markers and 8 SNPs, would however make this result not significant (p = 0.1) indicating the need for replication in other samples, especially from populations that have shown linkage to this region. No Bulgarian sample has been investigated for linkage to SZ, and we don't know if linkage to 5q is present in that population.

SMAD proteins are intracellular mediators in the bone morphogenetic proteins (BMPs) signalling pathway. SMADs are the only known BMP receptor substrates capable of signal transduction. Activated by phosphorilation SMADs move to the nucleus where they assemble complexes directly involved in the control of gene expression. *SMAD5 *is expressed in early differentiated granule neurons of the developing cerebellar cortex. BMP2 signalling activity is directly mediated by *SMAD5 *and its expression is sufficient to trigger granule cell precursor differentiation [46,47]. It can be speculated that dysfunctional *SMAD *genes can cause disruption in BMP signalling pathway which is directly linked to the transcriptional control of the oligodendrogenesis [48].

Defects in *TGFBI *are the cause of several types of corneal dystrophies. TGFBI protein binds to different types of collagen and is important for the cell- collagen interactions in cartilage. The function of the protein and its low expression in the brain make *TGFBI *a less plausible candidate gene for schizophrenia [49].

The second region includes rs6897690 and rs7443175, which are located in intron 1 and intron 2 in the *SPRY4 *gene. Indeed the signal we identify is not strong, but it points to a plausible candidate gene. *SPRY4 *encodes for a SPROUTY protein, which is widely expressed, including in brain. *SPRY4 *was identified as the evolutionarily conserved target of WNT/β-catenin signalling pathway involved in numerous processes during vertebrate CNS development.

It is possible that we have missed to detect a stronger association within the 5q31-33 region, as our coverage with microsatellite markers was still quite sparse and relied on assumptions about strong uniformly-spread LD. We have 13 intervals with intermarker distance over 300 kb due to the absence of polymorphic markers, or failure of the chosen ones to be analysed confidently. Therefore, we might have missed to detect the presence of a disease susceptibility locus due to the large distance exceeding the assumed presence of LD between any markers.

## Conclusion

In summary, our screening of the 5q31-32 linkage region using DNA pooling implicates three possible candidate genes: *SMAD5*, *TGFBI *and *SPRY4*. While none of the findings would survive correction for multiple testing, these results were found by a systematic analysis of one of the strongest schizophrenia linkage regions, making them good candidates to study in other samples, especially samples that have shown linkage to this region.

## Abbreviations

SZ = schizophrenia; SNPs = single nucleotide polymorphisms; Mb = megabase; kb = kilobase; LD = linkage disequilibrium; GWA = genome-wide association; TDT = transmission disequilibrium test; ETDT = extended transmission disequilibrium test; CNS = Central Nervous System.

## Competing interests

The author(s) declare that they have no competing interests.

## Authors' contributions

IZ carried out the laboratory work, did the statistical analysis and drafted the manuscript. LG constructed the pools, and together with GK supervised the laboratory work in Cardiff, helped in the statistical analysis and drafting the manuscript. DT supervised the laboratory work in Sofia and the recruitment of cases. GK and MJO recruited the patients. GK and IN made the diagnoses. IN developed the database used for storing and analysing phenotypic and genotypic data for the trios sample. GK, MJO and MCO'D critically evaluated the analysis and drafting of the manuscript. All authors read and approved the final manuscript.

## Pre-publication history

The pre-publication history for this paper can be accessed here:


